# pH-Sensitive Release of Functionalized Chiral Carbon Dots from PLGA Coatings on Titanium Alloys for Biomedical Applications

**DOI:** 10.3390/polym17192667

**Published:** 2025-10-02

**Authors:** Roberto López-Muñoz, Pascale Chevallier, Francesco Copes, Rafik Naccache, Diego Mantovani

**Affiliations:** 1 Science Faculty, Department of Chemistry, Sherbrooke University, Sherbrooke, QC J1K 2R1, Canada; jose.roberto.lopez.munoz@usherbrooke.ca; 2 Laboratory for Biomaterials and Bioengineering, CRC-I, Department of Min-Met-Materials Engineering & CHU de Québec Research Center, Regenerative Medicine, Laval University, Québec, QC G1V 0A6, Canada; pascale.chevallier@crchudequebec.ulaval.ca (P.C.); francesco.copes.1@ulaval.ca (F.C.); 3 Department of Chemistry and Biochemistry and the Centre for NanoScience Research, Concordia University, Montreal, QC H4B 1R6, Canada; rafik.naccache@concordia.ca; 4 Quebec Centre for Advanced Materials, Concordia University, Montreal, QC H4B 1R6, Canada

**Keywords:** Titanium alloys, PLGA coating, chiral carbon dots, surface modification, pH-responsive release

## Abstract

Titanium and its alloys are the most widely used metallic materials for bone contact implants. However, despite advances in implant technology, these alloys are still susceptible to post-operative clinical complications such as inflammation, which is often joined by infections and biofilm formation. A number of coatings were studied to overcome the drawbacks of these complications, but the controlled release of bioactive molecules over the first few days and the adhesion of the coating to the substrate remain recognized challenges. Carbon dots and the antibacterial potential of chiral carbon dots (CCDs) were recently reported, and their chirality was identified as a major contribution to the bactericidal effect. This study aimed to achieve a stimuli-responsive medium-term controlled release for up to one month. Two types of chiral carbon dots (CCDs) with distinct functional groups were incorporated into a stable and adherent biodegradable polymer coating, i.e., poly(lactic-co-glycolic acid) (PLGA). To enhance the coating adhesion, the titanium alloy surfaces were pre-treated and activated. The wettability, morphology, and surface composition of the coatings were characterized by contact angle, profilometry, SEM, and XPS, respectively. Coating degradation, adhesion, and CCDs release were studied at physiological pH (7.4) and at an acidic pH characteristic of an inflammatory site (pH 3.0) for up to one month. Their biological performances and blood compatibility were assessed as well. Degradation studies conducted over 28 days revealed a slow mass loss of approximately 10%, with maximum release rates for CCDs-OH and CCDs-NH_2_ of 67% and 45% at pH 7.4, respectively. At pH 3.0 an inverse trend was observed with 49% and 59% maximum release after 28 days. Furthermore, the coatings did not exhibit any cytotoxic and hemolytic effects. These findings demonstrate the potential of this approach to providing titanium implants with pH-sensitive controlled release of bioactive CCDs lasting up to one month, which could address key challenges in implant-associated complications.

## 1. Introduction

Metallic implants, particularly titanium and its alloys, are widely used for bone applications such as fracture fixation, dental implants, joint replacement, and spinal fusion, due to their high strength-to-weight ratio, durability, and biological performances. Titanium alloys also offer excellent corrosion resistance thanks to their passive titanium oxide layer, whose hydrophilic nature allows proteins to interact and cells to adhere to the surface [1]. The main factors influencing their long-term success are their integration with the bone, known as osseointegration, and the absence of inflammation or infection around the implant site. However, post-operative complications, including bacterial infections, still remain one of the leading causes of implant failure. For example, 4.3% of orthopedic implants fail each year due to bacterial infections in the US alone, and for dental implants, infection rates rise to 10–20% [2]. In addition, bacterial colonization, which often forms biofilms on the implant surface, is also known to inhibit the osseointegration process and therefore to impact the long-term success of the implant [3]. The other risk factor associated with infections is the inflammatory process of the tissues surrounding implants, which can then lead to a loosening of the implant and the need for revision surgery, which is painful for patients and an added economic burden for healthcare systems.

Over the last few decades, researchers have focused their attention on tackling these issues. As numerous in vivo studies demonstrate the influence of implant surface properties, surface roughness, surface free energy, and surface chemistry on the clinical success of implants [4,5,6], the proposed strategies are therefore mainly based on surface modifications as well as proactive and/or antibacterial coatings, achieved through different approaches [7,8]. For instance, by optimizing the surface roughness at around 0.2 μm through sandblasting, grit-blasting, acid-etching, or combination, osseointegration and antibacterial properties of Ti implants can be balanced [9,10]. However, despite reducing bacterial adhesion in the short term (from a few hours to a few days), the long-term problem remains. Therefore, coatings loaded with antibacterial agents have been of particular interest to researchers, since locally delivered antibacterial agents offer the advantage of a higher concentration directly at wound and infection sites.

In this context, the development of release-based antibacterial coatings has intensified with the emphasis on nanoparticles (NPs) such as Ag, CuO, ZnO, and TiO_2_ [11]. They offer improved diffusion capacity into the extracellular polymeric matrix once the biofilm has formed, and a broad spectrum of antibacterial activities that limit resistance, unlike antibiotics. While these NPs exhibit biocidal activity, they can induce cytotoxicity or pro-inflammatory responses for the surrounding tissues [12,13,14]. Recently, the emergence of carbon dots (CDs) as a promising class of antimicrobial agents has grown in the biomedical field due to their various advantages such as their low-cost preparation, the use of natural precursors (citric acid, simple sugars, or amino acids), their high biocompatibility, stability, versatile size (from 1 to 10 nm) and surface chemistry, as well as their lower propensity to induce bacterial resistance [15,16,17]. Of particular interest is that the broad spectrum of antimicrobial activity was confirmed against both Gram-positive and Gram-negative bacteria. Moreover, CDs and the duality between their synthesis sources and their functional groups, mainly OH, COOH, NH_2_, and SH, have also been shown to impact antimicrobial behavior, as shown by Hussen et al. [18].

Moreover, CDs with chiral properties have gained increasing attention due to their unique optical and biochemical characteristics. A recent and advanced search in SciFinder, Scopus, and Web of Science revealed that 40% of publications related to CCD are focused on optical, electron, and mass spectroscopy, while 53% are related to biochemical methods, ceramics, pharmaceuticals, and physicochemical properties. Only a small 7% of the publications concern the use of CCDs for antimicrobial applications, thus highlighting this area as potentially emerging. In addition, the chirality of CDs was shown to also affect their antibacterial efficacy, owing to improved selectivity, tunable optical properties, and targeted interaction with bacterial cells [19,20]. In fact, *D-cys*CDs displayed antibacterial properties against various bacteria strains at MICs, that were twice as low as those of *L-cys*CDs [21]. The same chiral source produced by different methods showed significant variations in MIC values against the same type of bacteria, with differences of up to 4- or 12-fold [22]. Song et al. demonstrated that *D-cys*CDs exhibit enhanced antimicrobial properties against Gram-positive bacteria and fungi, compared to *L-cys*CDs, when dual light irradiation with different wavelengths is applied [23].

One limitation regarding local delivery of antibacterial agents is their release, which must be controlled and, ideally, carried out on demand when inflammation and infection occur. For this reason, the composition of the coating is of major importance. Polymer-based coatings, particularly biodegradable ones [24], are attracting attention for their ability to control drug release while being biocompatible [25,26]. Among them, poly(lactic-*co*-glycolic) acid (PLGA) has emerged due to its properties including tunable mechanical properties, favorable degradation characteristics, wide range of release times (days to months), and high biocompatibility [27]. Indeed, the degradation of PLGA results in the release of lactic and glycolic acids, which are naturally present in the body and commonly figure as part of the various metabolic pathways. Moreover, PLGA has been shown to have low systemic toxicity, thus leading to FDA-approved implants. Additionally, the overall physical properties of the polymer-drug matrix can be optimized by controlling the polymer molecular weight and the ratio of lactide-to-glycolide [27,28,29]. In addition, PLGA is known to degrade faster in acidic conditions than in neutral pH ones, which means that when inflammation begins at the implantation site, the local pH decreases drastically (pH ~ 3), and the loaded drug will be released more rapidly [30]. Furthermore, Xu et al. demonstrated that both acidic and hydrophilic properties of the loaded drug were responsible for the enhanced PLGA degradation. In fact, hydrophilicity and acidity facilitated water adsorption and deprotonation of the PLGA matrix, respectively, both increasing the PLGA degradation rate [31].

Therefore, although the antibacterial potentials of CDs and CCDs are increasingly recognized, their integration into a PLGA matrix with TA and CaCl_2_ as additives, as well as their pH-responsive release behavior, remains unexplored. In this research, we addressed the design and the characterization of PLGA coatings loaded with CCDs bearing different terminal groups (–OH, –NH_2_). Additionally, to ensure stable and adherent coating over time, TA and calcium ions (Ca^2+^) were added to this formulation to promote anti-inflammatory and osseointegration properties [32,33]. Since the functionality of CCDs, as previously illustrated, influences their antibacterial properties, the main goal of this study was centered on the coating morphology, degradation behavior, pH-effects on release profiles, and cytocompatibility. By establishing a controlled release platform for CCDs under acidic and physiological conditions, this work is laying the foundation for the future development of infection-responsive medium-term implant coatings.

## 2. Materials and Methods

Tannic acid (TA), sodium hydroxide (≥97%), dopamine hydrochloride, Trizma^®^ base (≥99.9%), calcium chloride dihydrate (≥99.0%), and poly(D,L-lactide-*co*-glycolide) with a lactide-to-glycolide ratio of 75:25 and molecular weight of 66–107 kDa were used. A mixture of trisodium citrate dihydrate and citric acid was used to prepare acidic-buffered saline at pH 3.0 to mimic an inflammatory pH environment, while phosphate-buffered saline (PBS) at pH 7.4 was used to simulate physiological pH. All analytical-grade reagents were purchased from Sigma-Aldrich (Oakville, ON, Canada) and used without further purification. For all experiments, nanopure water (18.2 MΩ·cm) produced by a PURELAB^®^ Flex water purification system was used. Chiral carbon dots (CCDs) were kindly provided by collaborators at Concordia University, and their physicochemical characterization, along with selected properties, are summarized in Table 1 based on a previous publication.

### 2.1. Preparation of Ti6Al4V Samples

Ti_6_Al_4_V specimens were obtained from a titanium plate (ASTM B265 Grade 5, Rolled Alloys Inc., Laval, QC, Canada) with a thickness of 1 mm. The specimens were cut into 1 cm^2^ squares and cleaned using acetone, deionized water, and methanol in an ultrasonic bath for 10 min per solution. Manual polishing was performed by applying sequential treatment with silicon carbide wet/dry sandpaper (LECO Corporation, Mississauga, ON, Canada) with grit grades of 240, 320, 400, 600, and 800. Finally, all specimens were cleaned by repeating the initial cleaning procedure and dried with compressed air. They were either used on the same day or stored under vacuum for future use.

### 2.2. Activation and Dopamine-Surface Modification

Titanium surface activation was performed by immersing the specimens in 10 mL of 2M NaOH solution and placing them in an ultrasonic bath for 30 min to enhance dopamine grafting. The specimens were then rinsed twice with deionized water in an ultrasonic bath for 10 min and subsequently dried using a compressed air gun. Dopamine grafting was then carried out under dark conditions in a 12-well plate, with each sample immersed in 2 mL of dopamine solution (2 mg/mL in 100 mM Tris buffer, pH 8.6) for 24 h under gentle agitation. Afterward, the specimens were thoroughly rinsed with water and dried using a compressed air gun.

### 2.3. Carbon Dots Loading and Dip Coating on Metallic Surfaces

**Scheme 1:**
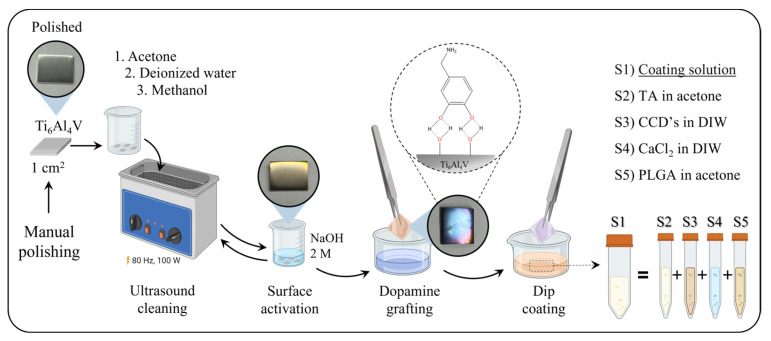
Schematic representation of the preparation process for titanium alloy sample pre-treatment and subsequent coating steps. For the coating solution S1, stock solution S3 containing CCD-OH was used for sample C6, while for sample C7, S3 was replaced with a new stock solution containing CCD-NH_2_.

Different dip-coating solutions, each with a total volume of 2 mL, were prepared for conditions C4, C5, C6, and C7, as outlined in Table 2 and represented in Scheme 1. These solutions were designed to evaluate their effects on the physicochemical and biological properties of the resulting coatings as well as the release behavior of the CCDs. For all conditions, dip-coating solutions were prepared using 100 mg/mL of PLGA dissolved in acetone. For conditions C5, C6, and C7, tannic acid (TA) and calcium chloride (CaCl_2_) stock solutions were prepared in acetone and water, respectively. The ratio of TA-to-CaCl_2_ in the dip-coating solutions was maintained at 1:1 to achieve a final concentration of 1 mg/mL. Additionally, for conditions C6 and C7, carbon dots functionalized with hydroxyl groups ([CCDs-OH]) and amine groups ([CCDs-NH_2_]) were dispersed in water and incorporated into the dip-coating solutions to reach a final concentration of 2 mg/mL. The amount of water used in the preparation of the dip-coating solutions was kept below 5% (*v*/*v*) to prevent the spontaneous hydrolysis of PLGA and phase separation.

A triple dip-coating process was applied to one side of the polished sample using 2 mL of the respective coating solutions. Each coating layer was allowed to dry for 30 s to ensure uniformity, resulting in a homogeneous coating with an approximate mass of 3 mg. All coated specimens were then dried under a controlled flow of compressed air at room temperature and stored under vacuum until use.

### 2.4. Surface Characterization

Surface roughness was measured using six specimens per condition by surface profilometry over a scan area of 1 mm^2^. These measurements were conducted with a Bruker Dektak XT Profilometer (Billerica, MA, USA) equipped with a 12.5 µm tip radius, a 65.5 µm range, and a stylus force of 1 mg. For the 3D map resolution, a trace spacing of 20 µm was used across a scan area of 4 mm^2^.

Surface morphology was analyzed using scanning electron microscopy (SEM) with energy-dispersive X-ray spectroscopy (EDX) (Quanta 250, FEI Company Inc., Thermo Fisher Scientific, Hillsboro, OR, USA). SEM micrographs were acquired under vacuum at low beam energy (3.0 kV) and a larger spot size (6.0) to prevent damage to the coating.

Contact angle measurements were performed using a VCA Optima XE system (AST Products, Billerica, MA, USA). Distilled water droplets of 0.5 µL were applied to each surface, with ten drops placed at different locations on three specimens per condition. The average of these measurements was reported as the contact angle value.

Chemical composition was examined using X-ray photoelectron spectroscopy (XPS) with PHI 5600-ci equipment (Physical Electronics, Chanhassen, MN, USA). Survey and high-resolution spectra were acquired at a detection angle of 45° and an analyzed area of 0.5 mm^2^, using a standard aluminum Kα X-ray source without charge neutralization. Three measurements for each sample were taken to confirm the homogeneity of the chemical composition. The curve fitting procedures were performed employing a least-square Gaussian–Lorentzian peak fitting procedure, after Shirley background subtraction. The C1s peaks were set at 285 eV (C-C and C-H) as reference.

Carbon dots dispersion was observed using a fluorescence microscope (LSM800 Axio Observer 7, Carl Zeiss, Canada Inc., Toronto, ON, Canada). Fluorescence images were obtained at 20X magnification.

### 2.5. Degradation Tests

Coating stability was evaluated over time in physiological media at two different pH values: pH 7.4 (PBS) and pH 3.0 (citrate buffer), representing an inflammatory environment. Briefly, coated specimens (C4, C5, C6, and C7) were placed in 12-well plates, and 3 mL of the respective medium was added to each well. All samples were tested in triplicate and were put into a water bath at 37 °C. Coating degradation was assessed on days 7, 14, 21, and 28 by weighing the specimens after they had been washed and dried under a controlled air flow for 2 h.

### 2.6. Release Profiles of Carbon Dots

To study the pH-dependent release of chiral carbon dots (CCDs) loaded into the PLGA matrix, specimens C6 and C7 were evaluated in triplicate over time in citrate buffer (pH 3.0) and phosphate-buffered saline (PBS, pH 7.4). At each time point (4 h, 8 h, 1 day, 3 days, 7 days, 14 days, 21 days, and 28 days), the medium was completely removed and replaced with an equivalent volume of fresh medium. The CCDs quantification was performed using fluorescent measurements (λ_exc_ 345 nm and λ_em_ 420 nm) with a plate reader (SpectraMax i3x, Molecular Devices, San Jose, CA, USA). The CCDs concentration was determined using a previously established calibration curve for each buffer solution (Figure S1). Results were analyzed and reported as the percentage of cumulative release.

### 2.7. Direct and Indirect Cytotoxicity Tests

Prior to cytotoxicity tests, the specimens Ti_6_Al_4_V (Ti), C5, C6, and C7 were polished and coated on both surfaces of the sample, then sterilized by UV irradiation using 2 cycles of 15 min of UV irradiation on both sides.

#### 2.7.1. Cell Culture

HDFs (human dermal fibroblasts) were cultured in Dulbecco’s modified Eagle’s medium (D-MEM) with 10% fetal bovine serum (FBS), penicillin (100 U/mL), and streptomycin (100 U/mL). The cells were maintained at 37 °C in a saturated atmosphere at 5% CO_2_. Media was changed every two days until 90–95% of confluence was reached. At this point, cells were detached from the plate using trypsin and then re-plated at a ratio of 1:5. Cells were used at passage 7 for the present experiments.

#### 2.7.2. Indirect Viability Assay

The indirect cytotoxicity test was performed following the ISO 10993-5:2009 procedure [34]. All samples were immersed in 660 µL of D-MEM culture medium, supplemented with 1% penicillin-streptomycin for 1, 3, and 7 days. At each time point, medium had been collected from samples and subsequently used for the viability test. One day prior to contact with the extract, HDFs were seeded into a 96-multi-well plate at a density of 20,000 cells/cm^2^ and incubated at 37 °C and 5% CO_2_ for 24 h in 100 µL/well of complete D-MEM. The day after, the medium was removed and 100 µL of the extracts were added to the well containing the cells and incubated for 24 h.

Cells cultured in complete D-MEM medium were used as a control condition (CTRL). Before putting them in contact with cells, extracted media had been supplemented with 10% fetal bovine serum (FBS). The extracts were then removed and 100 μL of 1% solution of resazurin sodium salt in complete D-MEM were added to the cells and incubated for 4 h at 37 °C and 5% CO_2_. After the incubation, the solutions containing the now-reduced resorufin product were collected and fluorescence intensity at λ_exc_ 545 nm/λ_em_ 590 nm wavelength was measured with a SpectraMax i3x Multi-Mode Plate Reader (Molecular Devices, San Jose, CA, USA). Fluorescence intensity is proportional to cell viability.

#### 2.7.3. Direct Viability Assay

The effect of the different coatings on cell viability has been analyzed using a direct viability assay performed using HDFs. Cells were seeded at a concentration of 20,000 cells/cm^2^ onto the different samples and incubated at 37 °C in a saturated atmosphere at 5% CO_2_. After 1, 3, and 7 days, the media was removed, and cells were incubated for 4 h with a 1X resazurin solution. After the incubation, the resorufin product obtained was collected and fluorescence intensity at λ_exc_ 545 nm/λ_em_ 590 nm wavelength was measured with a SpectraMax i3x Multi-Mode Plate Reader. Fluorescence intensity is proportional to cell viability.

### 2.8. Hemocompatibility Tests

Human blood from healthy donors had been collected in citrate-containing blood collection tubes. Three samples for each condition were placed in a 15 mL tube and 10 mL of sterile PBS 1X was added in each tube. PBS 1X was used as a negative control (CTRL Neg), and deionized H_2_O as a positive control (CTRL Pos). Samples and controls were incubated at 37 °C for 30 min. In the meantime, the collected blood was diluted in PBS 1X to a final ratio of 4:5 (4 parts of citrated blood and 5 parts of PBS 1X). After the incubation, 200 μL of diluted blood was added to each tube and carefully mixed by inverting each tube. Then, samples and controls were incubated at 37 °C for 1 h. All tubes were carefully mixed by inversion after 30 min of incubation. At the end of the incubation, the tubes containing the samples and the controls underwent a centrifugation step at 800× *g* for 5 min. The supernatant was collected, and 100 μL aliquots were placed in a 96-well plate. The absorbance (OD) at a wavelength of 540 nm was recorded and hemolysis calculated according to the following equation:

(1) $$Hemolysis\;\left(\%\right)\;=\;\;\;\frac{\;\;\;\;\;\;\;(ODsample-OD\;negative\;control)}{\;(ODpositive\;control-ODnegative\;control)}\times 100$$

### 2.9. Statistical Analysis

Statistical significance was calculated using the ANOVA non-parametric Kruskal–Wallis method with Dunn’s post-test through the software InStat™ (version 3.05). Values of *p* < 0.05 or lower were considered significant.

## 3. Results and Discussion

### 3.1. Surface Morphology and Wettability

**Figure 1:**
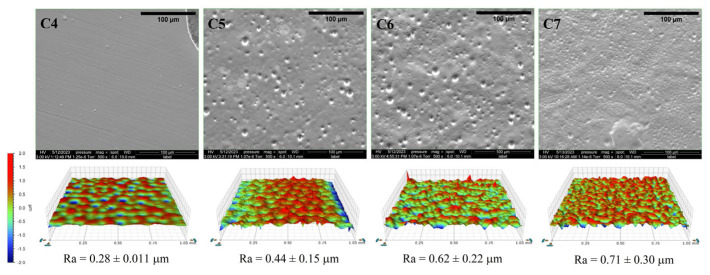
Surface morphology, topography, and roughness (Ra) after dip coating. Colored scale bar indicates the higher peaks (red zones) and deeper pores (blue zones). All SEM images have the same scale bar of 100 µm.

Since the implant’s surface properties influence the implant’s clinical success, the surface morphology, topography, and surface free energy were evaluated using SEM, profilometry (Figure 1), and water contact angle (Figure S2), respectively. First, PLGA coating morphology appeared to be smooth and homogeneous, as seen in the SEM images, with a roughness Ra of ~0.28 µm. However, the coating thickness was evaluated by a cross-sectional SEM image (Figure S3) and found to be around 4.8 ± 0.5 µm. The incorporation of TA/CaCl_2_ led to a slightly different morphology, with some valleys and pores, with an increase in Ra compared to only PLGA coating from 0.28 µm to 0.44 µm. The addition of CCDs, whatever their functionality, still showed the same morphology of valleys and pores as C5, however the roughness increased significantly: from 0.44 µm to 0.62 µm for CCDs-OH and 0.71 µm for CCDs-NH_2_, but with high standard deviation (around 0.2–0.3 µm) compared to PLGA. This could be due to the addition of water for CaCl_2_ and CCDs dispersion, even if the total amount of water used in the PLGA coating was kept ≤ 5% (*v*/*v*) to prevent degradation and phase separation. Additionally, the presence of valleys could be attributed to two possible factors: (i) the rearrangement of PLGA chains due to interactions with TA through hydrogen bonding between dopamine and carboxylic groups in PLGA; and (ii) the formation of circular valleys by solvent microdroplets (observed in SEM images for C5, C6, and C7), mainly caused by the rapid evaporation of acetone during the dip-coating process. Regarding the wettability, even if there were some differences in roughness, the WCA remained approximately the same at 80° ± 6° for the C4, C5, C6, and C7 samples, suggesting that the addition of TA/CaCl_2_ and the two types of CCDs did not significantly affect the coating’s wettability. This is likely because the PLGA used was rich in lactide (75%), which is known to be hydrophobic, typically exhibiting a WCA above 90°.

The influence of CCD functionalization on coating composition was also evaluated by FTIR analysis (Figure S4). The results revealed no additional peaks, particularly in the case of C6 sample compared to C5 sample (PLGA coating without CCDs), due to the overlap of the broad –OH and –COOH absorbance bands of PLGA (originating from lactic and glycolic acid units) with the hydroxyl groups from CCD-OH. Although PLGA does not contain amine groups, the characteristic N–H stretches from C7 loaded with CCD-NH_2_ were not clearly observed, probably due to the low CCDs concentration within the matrix.

### 3.2. Effect of [CCDs-OH]/[CCDs-NH_2_] Incorporation

**Figure 2:**
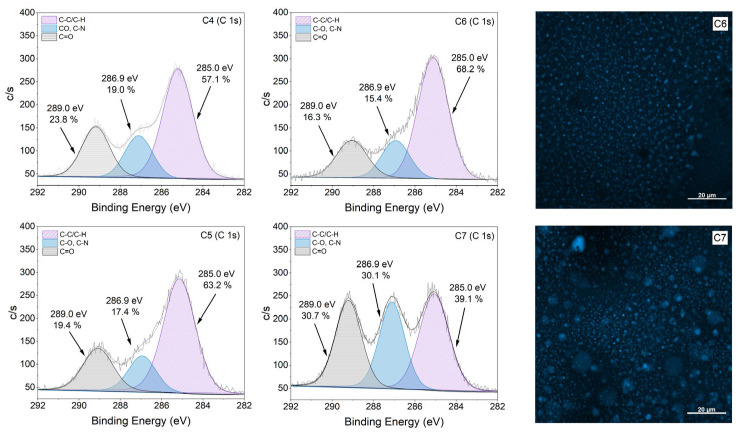
Deconvoluted high-resolution XPS spectra of C 1s and fluorescence images of PLGA coatings loaded with CCDs-OH (C6) and CCDs-NH_2_ (C7).

The XPS survey analysis of all the specimens confirms the efficiency of surface modifications from surface activation with NaOH, to dopamine grafting, and finally to the various PLGA-based coatings (Table S1). After the surface activation with NaOH, there were no significant changes in the surface composition. The dopamine grafting was clearly demonstrated by the disappearance of the Ti 2p peaks, the presence of 7.7 ± 0.6% N 1s peak, and the increase in the C 1s from 22.3 ± 2.8% (C2) to 68.3 ± 1.2% (C3). The PLGA coatings, independent from the sample composition, showed only C 1s and O 1s peaks, as expected due to their chemical composition, and, interestingly, the N 1s peak from dopamine was no longer detected. The effect of CCDs incorporation into the PLGA matrix was further investigated with high-resolution XPS (Figure 2). The C 1s peak for all the coated specimens were decomposed into three main peaks, assigned to: C-C and C-H (BE = 285.0 eV), C-O at ~286.9 eV, and O-C=O from carboxylic and ester groups 289.0 eV.

The increase in C-C/C-H band in C5 compared to C4, from 57.1% to 63.2%, can be attributed to the presence of TA, used for its anti-inflammatory properties and its ability to react with other components, either with dopamine on the Ti-surface or with the carboxylic groups from PLGA. Indeed, TA is rich in phenyl groups, but also hydroxyl groups (25/molecule) that will facilitate hydrogen bonding or electrostatic interactions [32,35]. A similar observation was reported by Zhang et al., who investigated the formation of a crosslinked coating on Ti surfaces using dopamine and TA via Michael addition/Schiff base reactions [36]. Similarly, the CCDs-OH incorporation also led to an increase in C-C/C-H peaks from 63.2% to 68.2%, and reduction in O-C=O from 17.4% to 16.3% and C-O/C-N from 17.4% to 15.4%, when compared to C5. Once again, this observation can be explained by the formation of hydrogen bonds due to the presence of oxygen-containing functional groups on CCDs-OH surface, as demonstrated by previously published XPS and FTIR analyses [21], which would interact with carboxylic groups of PLGA. Surprisingly, when CCDs-NH_2_ were added, the HR spectrum of C1s clearly showed a different behavior. Indeed, the atomic percentage of C-O/C-N (286.9 eV) and O-C=O (289.0 eV) increased significantly compared to C5, from 17.4% to 30.2% and 19.4% to 30.7%, respectively, while for CCDs-OH the contributions of these bands decreased. This could be explained by the presence of greater number of CCDs-NH_2_ on the outer surface of the coating, as the XPS depth analysis is approximately 5 nm depth. In addition, these CCDs-NH_2_ previously characterized by XPS exhibited characteristic peaks ascribed to amine and carboxylic acid moieties [17,37]. Moreover, the presence of -NH_2_ groups on the surface of the CCDs could promote surface modification and reactions with other polymer matrix components. These reactions may involve the incorporation of oxygen atoms from PLGA or TA into the CCDs structure, leading to the formation of C-O and O-C=O bonds. These effects could be caused by protonation and deprotonation from amide functional groups due to some CCDs having been reported to be sensitive to pH changes [38,39].

Fluorescence images were then taken to assess the dispersion/distribution of the CCDs within the coating (Figure 2, C6 and C7). The fluorescence images show that the CCDs were uniformly distributed in the PLGA matrix despite few small agglomerates. However, the CCDs-NH_2_ apparently exhibits a slightly more intense fluorescence, which could be attributed to the presence of a larger number of small agglomerates compared to CCDs-OH. It is interesting to note that the larger amount of CCDs-NH_2_ detected in fluorescence corroborated the XPS results. This increase in intensity and difference in cluster size difference could also be related to the protonation and deprotonation of the hydroxyl/carboxylic groups on the surface of the CCDs, caused by changes in the pH environment [40]. Indeed, Ehtesabi et al. [41] reported that some CCDs synthesized from citric acid and amide compounds undergo reversible protonation and deprotonation of carboxylic and amide groups, promoting pH changes that result in either a decrease or increase in fluorescence emission. Similarly, Sharma et al. [42] confirmed that fluorescence intensity responds to pH changes by affecting the energy levels of the CCDs, promoting aggregation and proton transfers.

### 3.3. Coating Degradation Behavior

**Figure 3:**
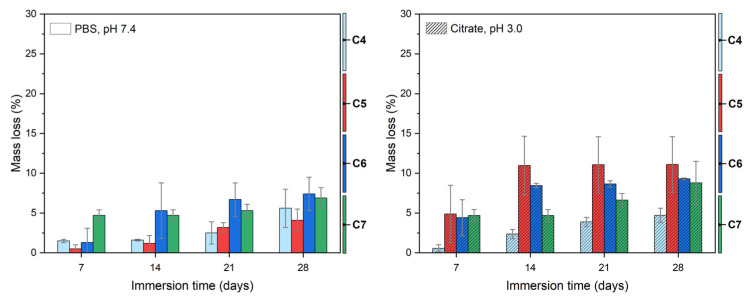
Coating degradation behavior in PBS (**left**) and citrate buffer saline (**right**) solutions at 37 °C for 28 days.

The coating degradation effect was closely investigated after incorporating TA, CaCl_2_, and CCDs into the PLGA matrix. PLGA was expected to exhibit a low degradation rate over time, regardless of the medium’s pH differences, due to its higher lactic acid content. This composition reduces susceptibility to hydrolysis by hindering water molecule attack [43]. As shown in Figure 3, PLGA displayed a maximum degradation of 5% ± 2% after 28-day incubation at both pH levels. The incorporation of TA and CaCl_2_ into the coating formulation was designed to achieve a synergistic effect, ensuring stability through electrostatic or hydrogen bond interactions from TA and enabling hydroxyapatite formation (via calcium ions) to support osteogenesis over time [43,44,45]. This incorporation did not significantly alter PLGA degradation at physiological pH compared to C4. However, under acidic conditions, maximum degradation reached 11% ± 3% after 28 days. This increase may be linked to protonation of TA’s pyrogallol groups that may potentially accelerate the PLGA’s hydrolytic degradation [32,46]. Additionally, CaCl_2_ may contribute to this degradation by increasing water uptake via electrostatic interactions between calcium ions and PLGA’s carboxylic groups, thereby promoting water diffusion and accelerating degradation [47].

Regarding CCDs incorporation, coatings containing C6 and C7 exhibited accelerated degradation compared to pristine PLGA coatings, particularly under acidic pH. Similar pH-responsive hydrolysis of PLGA has been reported in composite systems containing hydrophilic additives or nanoparticles [48,49], where water uptake facilitates ester bond cleavage. These findings are consistent with the literature, but it can be noted that despite the different terminal groups, C6 and C7 displayed nearly identical degradation rates. This contrasts with earlier reports showing clear functional group-dependent degradation kinetics [43], suggesting that in this system the dominant factor may be the overall hydrophilicity introduced by CCDs incorporation rather than specific chemical functionality. Thus, our results extend previous findings by showing that the balance of hydrophilicity between –OH- and –NH_2_- decorated CCDs can converge to similar macroscopic degradation outcomes within a PLGA matrix.

At pH 7.4, C6 and C7 showed ~ 7% ± 1% mass loss after 28 days. In contrast, at pH 3.0, degradation followed a similar pattern but reached ~ 9% ± 1% mass loss for both formulations under the same timeframe. A similar behavior was observed by Sahiner et al., who used nitrogen-doped carbon dots embedded in PLGA films. These films promoted a proportional release corresponding to increased carbon dot concentration, influenced by PLGA film degradation [48]. On the other hand, the similar degradation behaviors observed for C6 and C7, despite differences in their terminal groups, can be explained by two factors. First, both coatings contain the same CCD loading, and at this relatively low concentration the effect of terminal chemistry on bulk PLGA hydrolysis is limited. Second, both –OH and –NH_2_ groups increase the overall hydrophilicity of the coating and promote hydrogen bonding with water molecules. Although –OH groups typically form stronger hydrogen bonds due to the higher electronegativity of oxygen compared to nitrogen, both functional groups can facilitate water uptake and spreading. This is consistent with our morphological analysis (Figure 1) and contact angle (Figure S3), which shows comparable increases in surface roughness for both coatings and similar wettability, promoting similar rates of PLGA matrix hydration and degradation.

The slightly accelerated degradation of coatings C5, C6, and C7 over time, compared with PLGA alone, may also be due to increased water absorption as a result of the higher roughness of these samples. After seven days of incubation, superficial defects and minor coating detachment were observed in SEM micrographs. Chen et al. also observed that by incorporating hydrophilic CCDs in PLA films, the roughness was increased, which in turn promoted water spreading [50].

### 3.4. Carbon Dots Release Profiles

**Figure 4:**
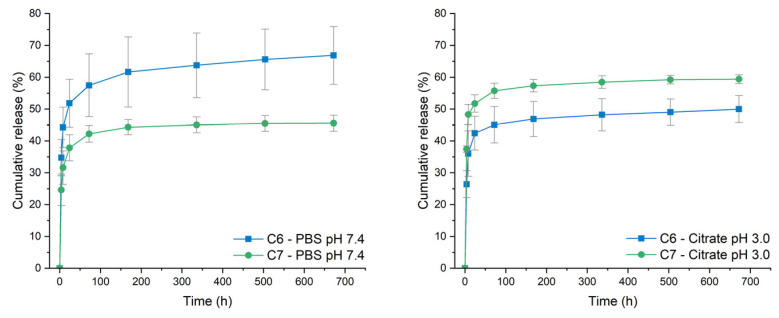
Cumulative release profiles of CCDs with hydroxyl groups (C6) and amino groups (C7) from PLGA matrix with TA and CaCl_2_ at pH 7.4 (**left**) and pH 3.0 (**right**).

The pH-dependent behavior of the CCDs with different surface functional groups has been extensively studied, but their release behavior from PLGA coatings is poorly described. Interestingly, the release behavior presented in Figure 4 shows an inverse effect between the CCDs with -OH and those with -NH_2_, depending on the pH of the release medium. At pH 7.4, the C6 samples exhibited a release of 57% ± 10% after 3 days, reaching a maximum of 67% ± 9% after 28 days. In contrast, the C7 samples showed slower release kinetics, with 42% ± 2% release after 3 days and a maximum of 45% ± 2% after 28 days. Conversely, under acidic conditions at pH 3.0, the release behaviors were reversed: the C7 samples displayed a release of 56% ± 2% after 3 days and a maximum of 59% ± 1% after 28 days, while the C6 samples showed a release of 45% ± 6% after 3 days and a maximum of 49% ± 4% after 28 days.

These release behaviors under neutral and acidic conditions suggest that the deprotonation and protonation of the surface functional groups on the CCDs may play an important role in controlling the release. The reduced release profile in CCDs functionalized with -OH and-COOH groups (C6) may stem from the protonation of these groups (-OH → -OH_2_^+^/-COOH → -COOH_2_^+^) in acidic media [51]. Under such conditions, protonation of hydroxyl and carboxyl groups on the CCDs surface likely diminishes their negative charge or renders them neutral, which leads to a reduction in electrostatic repulsion that could promote aggregation [52], thereby slowing release dynamics. In contrast, at neutral pH, these functional groups remain predominantly deprotonated (-O^−^), enhancing their negative surface charge. The increased electrostatic repulsion improves water dispersibility, which may facilitate a more sustained or efficient release profile. On the other hand, although the 14% increase in release for amine-functionalized CCDs at pH 3.0 compared to pH 7.4 may not be statistically significant, the protonation of amino groups (-NH_2_ → -NH_3_^+^) in acidic conditions likely generates a strong positive surface charge [53]. This charge dominance could enhance colloidal stability through electrostatic repulsion between particles, potentially explaining the slight pH-dependent release behavior.

### 3.5. Biocompatibility

**Figure 5:**
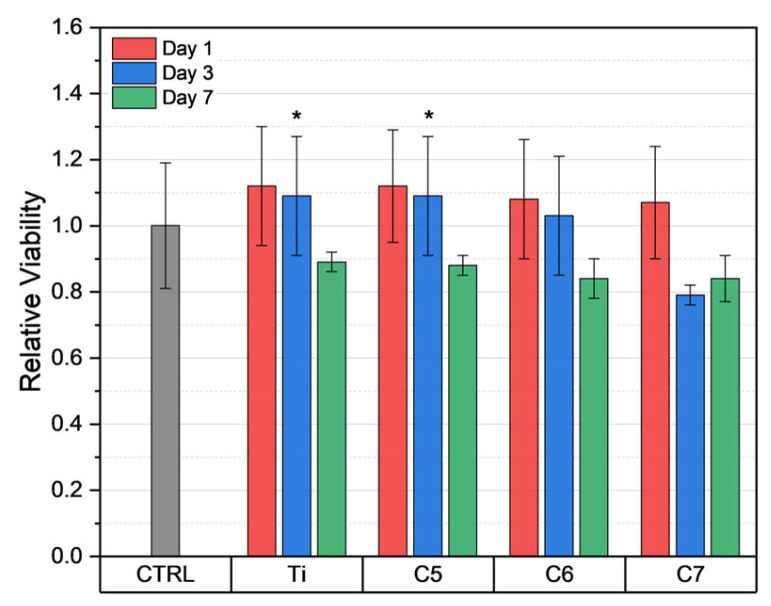
Indirect Cytotoxicity Assay. HDFs were treated with the following samples: standard culture medium (CTRL); extracts from: (1) Ti6Al4V (Ti); (2) C5; (3) C6; and (4) C7. Cell viability was measured after 1 day of incubation by means of a resazurin salt solution assay. Results have been normalized against the CTRL condition. The bar chart shows the mean relative viability ± SD recorded from HDFs treated with different experimental conditions. * *p* ˂ 0.05 vs. C7 Day 3.

Indirect cytotoxicity tests were performed using HDFs. As shown in Figure 5, the extracts obtained from all the experimental conditions tested did not significantly alter cell viability compared to the CTRL condition. Moreover, no differences were noted in between the experimental conditions. As for the extracts from day 3, once again the extracts obtained from the different experimental conditions did not exert any effects on cell viability when compared to the CTRL condition. However, the viability measured with the C7 extract was significantly reduced when compared to both the Ti and C5 conditions (*p* ˂ 0.05). Although no significant differences in cell viability were observed between groups on day 7, all conditions exhibited a modest decrease compared to day 3. This reduction can be explained by two factors. First, PLGA-based coatings are known to exhibit an initial burst-release phase during early degradation, which can transiently influence local microenvironments and thereby affect cell proliferation [27,54]. Second, by day 7, cell cultures on all substrates including titanium controls approached confluence, where nutrient depletion and contact inhibition naturally limit further proliferation [55]. Importantly, even with this decrease, viability remained within ranges typically considered acceptable for cytocompatibility, supporting the biocompatible nature of the coatings.

**Figure 6:**
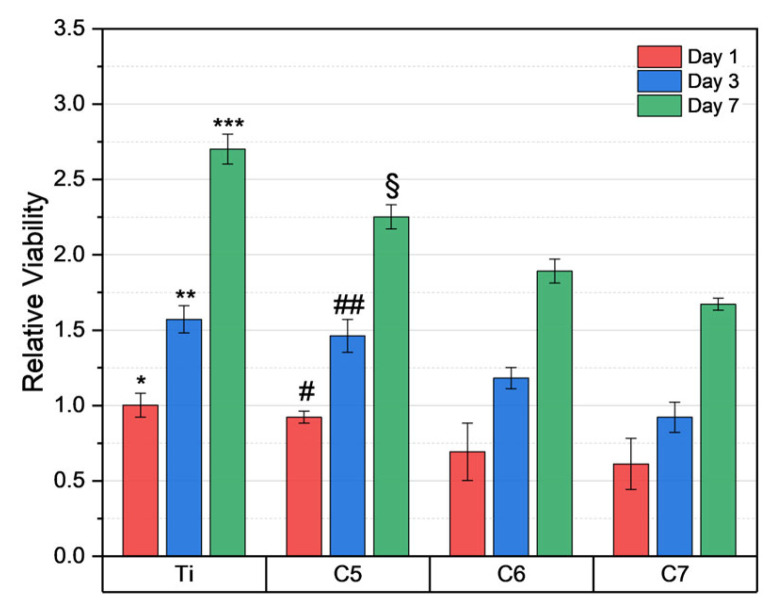
Direct Viability Assay. HDFs were directly seeded on the following samples: standard culture medium (CTRL); extracts from: (1) Ti6Al4V (Ti); (2) C5; (3) C6; and (4) C7. Cell viability was measured after 1, 3, and 7 days of incubation by means of a resazurin salt solution assay. Results have been normalized against the Ti condition. The graphic shows the mean relative viability ± SD recorded from HDFs seeded on the different samples. * *p* ˂ 0.001 vs. C6 and C7 Day 1; # *p* ˂ 0.05 vs. C6 Day 1 and *p* ˂ 0.001 vs. C7 Day 1; ** *p* ˂ 0.01 vs. C6 Day 3 and *p* ˂ 0.001 vs. C7 Day 3; ## *p* ˂ 0.001 vs. C7 Day 3; *** *p* ˂ 0.001 vs. C6 and C7 Day 7; § *p* ˂ 0.001 vs. C7 Day 7.

Regarding the direct cytotoxicity test, the results are presented in Figure 6. After 1 day of incubation, both the Ti and C5 conditions showed a significantly higher cell viability compared to both the C6 (*p* ˂ 0.001 for Ti and *p* ˂ 0.01 for C5) and C7 (*p* ˂ 0.001 for both Ti and C5) conditions. No differences were noted in between the Ti and C5 conditions, nor between the conditions of C6 and C7. On day 3, the Ti condition showed significantly higher viability compared to the C6 (*p* ˂ 0.01) and C7 (*p* ˂ 0.001) conditions, but once again there were no significant differences compared to the C5 condition. As for the C5 condition, the viability of the HDFs seeded on these samples were significantly higher compared to the C7 condition (*p* ˂ 0.001). Finally, after 7 days of incubation, once again the cells seeded on the Ti samples showed a significantly higher viability compared to both the C6 and C7 conditions (*p* ˂ 0.001). This effect can be attributed to the release of CCDs from the PLGA matrix during the early degradation phase. While CCDs are generally reported as biocompatible, high local concentrations have been shown to transiently influence cell adhesion, oxidative balance, or proliferation dynamics without causing acute cytotoxicity [56,57,58]. The modest reduction observed in our system likely reflects this effect and did not compromise overall cytocompatibility, as viability values remained within acceptable ranges throughout the study. These findings suggest that CCD incorporation may slightly modulate cell growth kinetics, while maintaining compatibility with mammalian cells. As for the latter, the viability recorded for C5 samples were significantly higher compared to the C7 condition (*p* ˂ 0.001). However, despite the significant differences reported, the cells seeded on both the C6 and C7 conditions showed progressive growth over the studied period of 7 days, demonstrating how both conditions are compatible with cell attachment, growth, and survival.

### 3.6. Hemocompatibility

**Figure 7:**
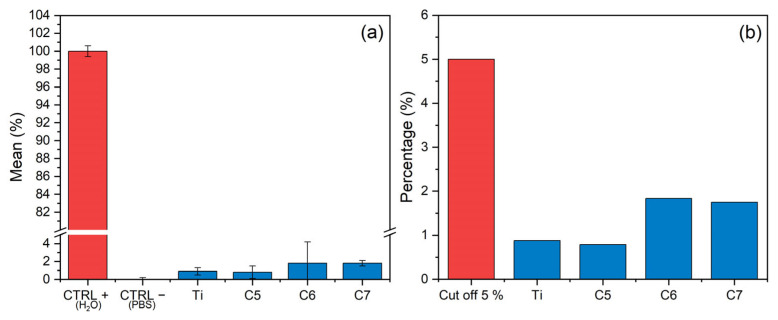
Hemolysis tests of Ti6Al4V as a reference, coated samples with and without CCDs. (**a**) shows the results of the hemolysis test performed on the different conditions compared to the positive control (CTRL Pos) and negative control (CTRL Neg) conditions, (**b**) shows the differences between the tested samples and the 5% hemolytic cut-off.

As for the hemocompatibility of the samples studied, the results of the performed hemolysis test (Figure 7a,b) show how the different coatings performed on the Ti surfaces do not induce hemolysis. In fact, for a material to be considered hemolytic, it must induce a hemolysis percentage higher than 5% (Figure 7b); hence the tested samples are not hemolytic. No significant differences were noted between the different conditions.

## 4. Conclusions

In this study, titanium alloy surfaces were coated with CCDs loaded with PLGA coatings, and their stability and degradation behaviors were evaluated, as well as the release behavior of CCDs as a function of pH. Finally, cytocompatibility on human cells and hemocompatibility were investigated. Homogeneous PLGA-based coatings were obtained even when CCDs, regardless of their function being either -OH/-COOH or -NH_2_, were added and the roughness remained low (less than 1 µm), as seen by SEM and profilometry analyses. In addition, fluorescence images showed that the CCDs were distributed uniformly within the coating, with larger numbers of CCDs-NH_2_ on the surface compared to CCDs-OH. Furthermore, thanks to the multi-step surface pre-treatment, from manual polishing to NaOH activation and dopamine grafting, PLGA coatings were demonstrated to be adherent and stable for up to 28 days regardless of pH. The release behavior of the CCDs was found to be dependent on the pH and terminal functionality of the CCDs. Indeed, pH 7.4 promoted a maximum release of 67% for CCDs-OH and 45% for CCDs-NH_2_, while at pH 3.0, the trend was reversed with 49% for CCDs-OH and 59% for CCDs-NH_2_. This could be explained by the ability of the terminal functionality of CCDs to be charged as a function of pH. Hydroxyl and carboxylic groups with a pKa below 5 are protonated at neutral pH, while -NH_2_ groups (pKa ≥ 7) are deprotonated.

Regarding the biological tests, cytocompatibility assays confirmed that CCD-loaded coatings maintained an acceptable level of cell viability (85%), with no evidence of cytotoxicity compared to control PLGA or titanium substrates. A slight reduction in proliferation was observed at intermediate time points, which can be attributed to transient CCD release, but viability remained comparable among groups on day 7. These results indicate that the integration of CCDs into PLGA coatings does not compromise overall biocompatibility, thereby supporting their potential for further antimicrobial evaluation.

Therefore, these results demonstrated a pH-responsive PLGA coating system capable of releasing chiral carbon dots under acidic conditions relevant to implant-associated infections. Although the antibacterial efficacy of the coatings themselves remains to be directly assessed, our results establish the feasibility of integrating CCDs into biodegradable polymer coatings to achieve controlled release and maintain cytocompatibility. This platform thus represents an important step toward the development of responsive implant coatings. Ongoing work will focus on direct antimicrobial validation in vivo, as well as clarifying the role of CCD chirality in infection-relevant environments.

## Figures and Tables

**Table 1:** Summary of key physicochemical and biological properties of cysteine-derived CCDs (from Victoria et al., 2020) [21].

Properties	Obtained Parameters	Technique
Average particle size	~12 ± 3 nm (range 6–28 nm)	TEM
Morphology	quasi-spherical, uniform distribution	TEM
Optical response	absorption peaks at ~250 and 350 nm	UV-vis
Fluorescence	*L-* and *D-cys*CDs were at λ_exc_ = 350 nm	PL-spectroscopy
Quantum yield	~14% (relative to quinine sulfate)	Photoluminescence
Chirality	mirror-image CD spectra for L- and D-cysCDs; distinct from precursors	Circular dichroism
Surface chemistry	–NH_2_, –COOH, –OH, S-containing groups	FTIR, XPS
Structure	predominantly amorphous at ~17° 2θ and sharp crystalline peaks	XRD
Thermal behavior	2% loss from 30 to 100 °C, 5% loss from 100 to 150 °C, and 58% loss between 200 and 500 °C	TGA
Antibacterial activity	4 mg/mL in *E. coli* for *L-cys*CDs 2 mg/mL in *E. coli* for *D-cys*CDs 2 mg/mL in *M. luteus* for *L-cys*CDs 0.5 mg/mL in *M. luteus* for *D-cys*CDs	MIC assays

**Table 2:** Different treatment conditions on Ti_6_Al_4_V surface.

Specimen	Type of Treatment
C0	Untreated Ti_6_Al_4_V
C1	Ti-polished (manual polishing)
C2	Ti-OH (activation with NaOH)
C3	Ti-Dopa (dopamine grafting)
C4	Ti-Dopa-PLGA
C5	Ti-Dopa-PLGA/TA/CaCl_2_
C6	Ti-Dopa-PLGA/TA/CaCl_2_-[CCDs-OH]
C7	Ti-Dopa-PLGA/TA/CaCl_2_-[CCDs-NH_2_]

## Data Availability

All data are contained within the article.

## Supplementary Materials

The following supporting information can be downloaded at: https://www.mdpi.com/article/10.3390/polym17192667/s1, Figure S1: Calibration curves of CCDs-NH_2_ and CCDs-OH in PBS pH 7.4 and citric acid pH 3.0 with a fluorescence reading at 420 nm; Figure S2: Effect of contact angle on different surface treatment conditions (significance 5%); Figure S3: SEM image of the coating thickness; Figure S4: FTIR of PLGA matrix and coated samples with CCD-OH (C6) and CCD-NH2 (C7); Figure S5: Thermogravimetric analysis of raw PLGA, C6, and C7; Table S1: Atomic percentages for each type of treatment on the Ti_6_Al_4_V surface.
