# Supplementary material for: pH-Sensitive Release of Functionalized Chiral Carbon Dots from PLGA Coatings on Titanium Alloys for Biomedical Applications
Source: Polymers (Basel). 2025 Oct 2;17(19):2667. doi: 10.3390/polym17192667 (PMC12526717; doi:10.3390/polym17192667)
Supplement: Supplementary file 1 [file polymers-17-02667-s001.zip › polymers-3872060-supplementary.pdf]

Supporting Information

Roberto López-Muñoz [1,2], Pascale Chevallier [2], Francesco Copes [2], Rafik Naccache [3,4] and Diego Mantovani [2,*]

[1] Science Faculty, Department of Chemistry, Sherbrooke University, Sherbrooke, QC J1K 2R1, Canada; jose.roberto.lopez.munoz@usherbrooke.ca

[2] Laboratory for Biomaterials and Bioengineering, CRC-I, Department of Min-Met-Materials Engineering. & CHU de Québec Research Center, Regenerative Medicine, Laval University, Québec, QC G1V 0A6, Canada; pascale.chevallier@crchudequebec.ulaval.ca; francesco.copes.1@ulaval.ca, diego.mantovani@gmn.ulaval.ca

[3] Department of Chemistry and Biochemistry and the Centre for NanoScience Research, Concordia University, Montreal, QC H4B 1R6, Canada; rafik.naccache@concordia.ca

[4] Quebec Centre for Advanced Materials, Concordia University, Montreal, QC H4B 1R6, Canada

* Correspondence: diego.mantovani@gmn.ulaval.ca

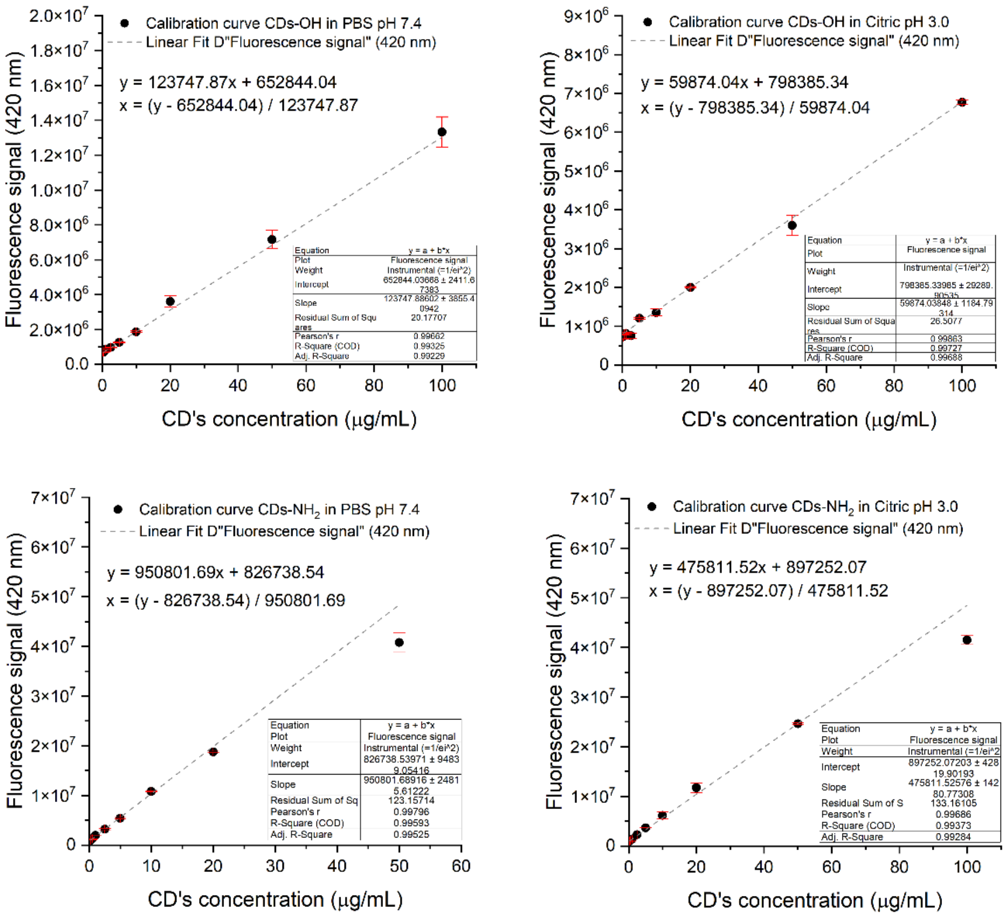

Figure S1. Calibration curves of CCDs-NH_2 and CCDs-OH in PBS pH 7.4 and citric acid pH 3.0 with a fluorescence reading at 420 nm.

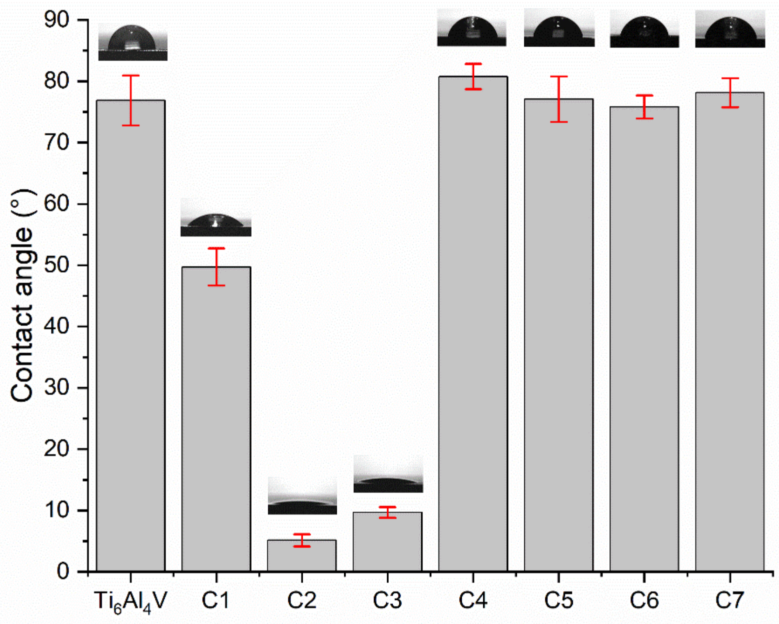

Figure S2. Effect of contact angle on different surface treatment conditions (significance 5 %).

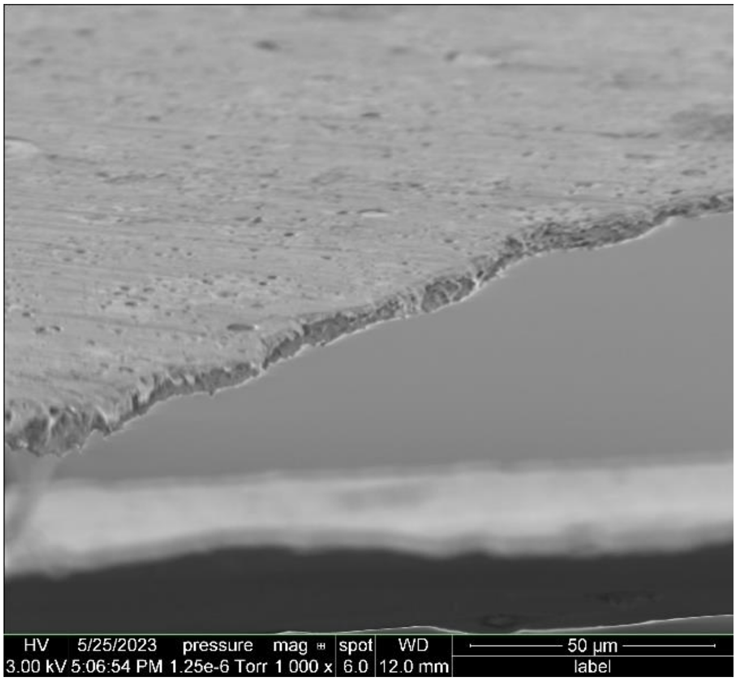

Figure S3. SEM image of the coating thickness.

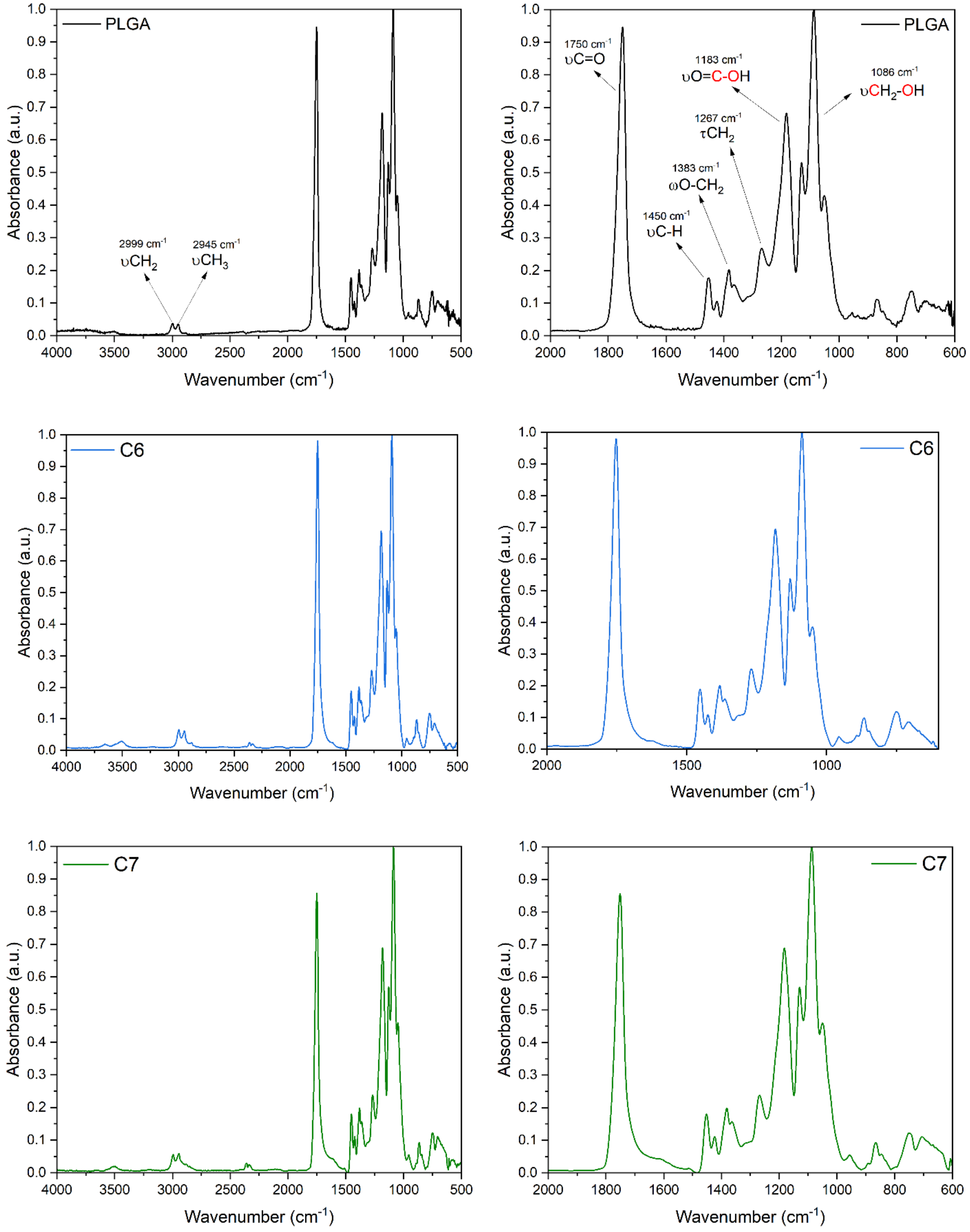

Figure S4. FTIR of PLGA matrix and coated samples with CCD-OH (C6) and CCD-NH_2 (C7).

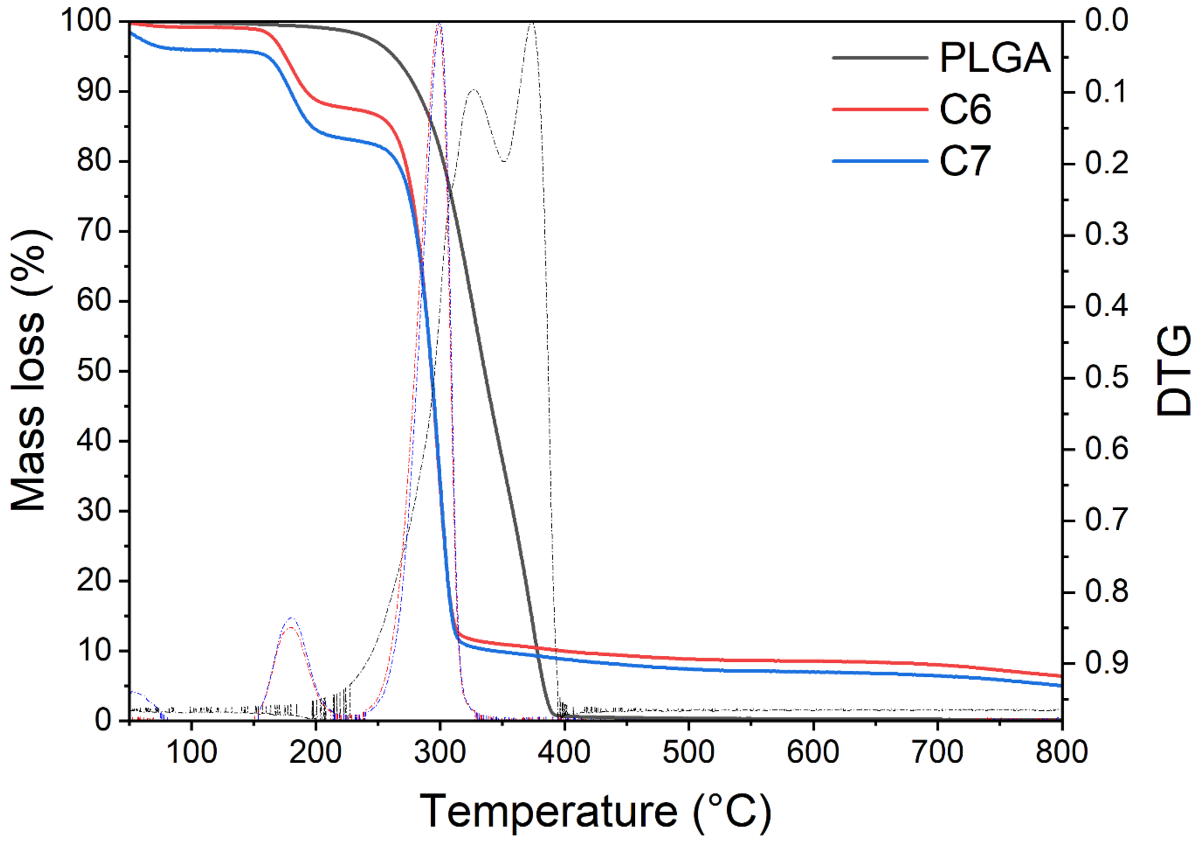

Figure S5. Thermogravimetric analysis of raw PLGA, C6, and C7.

Table S1. Atomic percentages for each type of treatment on the Ti_6Al_4V surface.

Samples	Atomic percentage				
	**% C**	**% O**	**% N**	**% Ca**	**% Na**
C1 (Ti-polished)	26.0 ± 2.9	57.0 ± 3.0	-	-	-
C2 (Ti-OH)	22.3 ± 2.8	50.7 ± 1.0	-	1.15 ± 0.6	8.6 ± 1.8
C3 (Ti-Dopa)	68.3 ± 1.2	22.7 ± 0.8	7.7 ± 0.6	-	-
C4 (Ti-Dopa-PLGA)	68.4 ± 0.2	31.5 ± 0.2	-	-	-
C5 (Ti-Dopa-PLGA-TA/$CaCl_2$)	71.3 ± 2.3	28.7 ± 2.3	-	-	-
C6 (Ti-Dopa-PLGA-TA/$CaCl_2$-[CCDs-OH])	72.8 ± 0.5	27.2 ± 0.5	-	-	-
C7 (Ti-Dopa-PLGA-TA/$CaCl_2$-[CCDs-NH_2])	63.5 ± 1.4	36.0 ± 1.4	-	-	-
